# Retrospective serological study of *Rickettsia* spp. and *Borrelia* spp. antibodies in patients with peripheral facial nerve palsy

**DOI:** 10.1080/20008686.2021.1987058

**Published:** 2021-10-25

**Authors:** Katarina Wallménius, Carl Påhlson, Kenneth Nilsson

**Affiliations:** a Department of Medical Science, Section of Clinical Microbiology, Uppsala University, Uppsala, Sweden; b Department of Medical Science, Section of Infectious Diseases, Uppsala University, Uppsala, Sweden

**Keywords:** Spotted fever rickettsia, facial palsy, serology, western blot

## Abstract

In a retrospective study, 36 patients with peripheral facial palsy were serologically evaluated for the presence of *Rickettsia* spp. and *Borrelia* spp. antibodies. All sera underwent immunofluorescence and Western blot analysis for IgG and IgM antibodies using *Rickettsia helvetica* and *R. felis* as antigens. Anti-*Borrelia* antibodies were detected using a commercial ELISA detecting *Borrelia burgdorferi, B. afzelii* and *B. garinii*. Three patients (8.3%) were seropositive for *Rickettsia* spp. with IgG titres equal to 1:128, and six patients (16.7%) had IgM titres equal to or above 1:128. All samples with IgG/IgM titres equal to or above 1:128 were confirmed by Western Blot. Four patients (11.1%) had IgG antibodies against *Borrelia* at a titre level normally judged to be indicative of current infection. Two of these patients had significant IgG or IgM titres for both *Rickettsia* spp. and *Borrelia* spp., indicating co-infection. In conclusion, the findings indicate current rickettsial infection or early response at about the same degree as for Lyme borreliosis in patients with facial palsy, but they need to be further examined with a larger number of patients and paired serum analyses.

## Introduction

An acute spontaneous unilateral peripheral paralysis of the facial nerve is characterized by muscle weakness on one side of the face [1]. About 3,000 people are diagnosed annually in Sweden. Most patients recover completely, but about 30% suffer sequelae of varying degrees. In two-thirds of the cases, the cause is unknown, i.e., Bell’s palsy. In other cases, where a cause of the paralysis is found, an association has been described between facial palsy and otitis media as well as between facial palsy and viral infections, such as varicella zoster, herpes simplex viruses or sometimes sarcoidosis [2]. A non-specific immunological response to infection, leading to facial nerve compression and degeneration, has been suggested as the pathogenic mechanism.

Lyme borreliosis (LB) is also accepted to be a cause of acute peripheral facial nerve palsy (FNP), and LB has been demonstrated in approximately 10–20% of patients in Sweden [3,4]. The correlation between FNP and neuroborreliosis is stronger, especially among children [5–7].

Moreover, several studies have reported that *Rickettsia* spp. are a cause of FNP, and in a previous study, we demonstrated seroconversion to *Rickettsia* spp. in 8.3% patients in Sweden with Bell’s palsy, together with PCR findings, indicating a possible causal relationship [8–11]. Besides an occasional reported finding of *R. sibirica, R. helvetica* is the only tick-transmitted rickettsia in Sweden, occurring in approximately 9% of *Ixodes ricinus* ticks [12]. Another spotted fever rickettsia, *R. felis*, the vector of which is cat fleas (*Ctenophalides felis)*, has been found in Sweden in a few patients with meningitis and in two patients with facial palsy, where *R. felis* was detected by performing PCR on the cerebrospinal fluid [9,13]. Although the rickettsial infection is often benign and associated with fever, the basic pathogenic mechanism is disseminated vasculitis, which may result in the involvement of many organs, including the nervous system [14]. Infections of the central nervous system (CNS), most commonly meningitis, encephalitis and disseminated encephalomyelitis as well as facial palsy or cerebral infarction, are known for 12 of the species [15,16].

The aim of the present study was to evaluate the seroprevalence of *Rickettsia* spp. and *Borrelia* spp. antibodies in patients diagnosed with FNP in relation to solitary infections and coinfections as well as the importance of the season.

## Patients and methods

### Patients

Previously stored and frozen material of serum samples from patients who had been diagnosed with FNP and sampled for analysis of *Borrelia* antibodies from January to June 2015 in the Department of Clinical Microbiology, Uppsala County, Sweden, were retrospectively examined for the presence of antibodies to *Rickettsia* spp. In total, 36 patients were included in the study (21 women and 15 men; median age 42 years, range 19–77 years). The patients had all visited a health-care centre or examination between 2 days and 36 weeks (in one exceptional case 4 years) after the onset of FNP. One patient had also been sampled for varicella zoster virus (VZV) DNA in skin blisters. None of the other patients had been tested for any viral infection (e.g., herpes, VZV). As a control group, sera from 50 healthy blood donors were chosen. The study was reviewed and approved by the Ethics Committee at Uppsala University (reg. no. 2016/152). The relatively limited material for the study was chosen as a pilot study, given that there was previous ethical approval for the collected material and that collecting more material on adults with facial paresis takes time. This is because sampling for *Borrelia* spp. in relation to this indication is rare. In addition, when making a clinical diagnosis, typically only individual serum samples are taken for analysis.

### Serology

*R. helvetica*, isolated from an *Ixodes ricinus* tick in Sweden [6] and cultured in Vero cells (ATCC33), was used as antigen for immunofluorescence (IFA). Moreover, *R. felis*, a gift from Dr J. Stenos, Australian Rickettsial Reference Laboratory in Geelong Australia, was used for the same purpose. Antigen and microscope slides were prepared and samples analysed as previously described and evaluated for both IgG and IgM antibodies in serum dilutions 1:64, 1:128 and 1:256 [17]. Fluorescein isothiocyanate-conjugated (FITC) ɣ- and mu-chain-specific polyclonal rabbit anti-human IgG and IgM (Dako, Denmark, ref. no. F202 and F203) were used as secondary antibodies for detection of IgG and IgM antibodies, the latter after pretreatment with rheumatoid factor adsorbent IgG/RF STRIPPER (The Binding Site Group Ltd, Birmingham, UK). Patient serum with 1:128 in end-point titre level for IgG and 1:256 for IgM was used as a positive control. The negative control was a human blood donor serum. The method is part of both the national reference diagnostics for rickettsia in Sweden and the quality assured routine diagnostics used at the Department of Clinical Microbiology at Uppsala University Hospital. All three investigators blindly evaluated all IFA slides, and the results are based on at least two of the three making consistent assessments. Laboratory evidence of current or previous infection with *Borrelia burgdorferi, B. afzelii* and *B garini* was based on analysis of serum samples conducted at the Clinical Microbiological Laboratory at Uppsala University Hospital, Uppsala, Sweden, using a commercial enzyme-linked immuno-sorbent assay (ELISA), according to the manufacturer´s instructions (Euroimmun AG, Lübeck, Germany). Values < 16 RU/mL were judged to be negative, 17–22 RU/mL borderline and >22 RU/mL positive.

*Western Blot* Sera from nine of the IgG/IgM positive patients (Pat. Nos. 1, 32, 33, 3, 8, 10, 17, 20 and 22) were diluted 1:200 and tested for Western Blot (WB) with *R. helvetica* whole-cell antigen using Amersham™ WB System (GE Healthcare) with the secondary antibody Anti-human IgG DyLight™549 and Anti-human IgM DyLight™ 549 (Rockland Inc. cat.no 609–142-123 and 609–142-007), in a titre level of 1:10,000 according to the manufacturers protocol. As a positive control, serum from a patient with a proven end-point IgG/IgM titre level of 1:256/1:128 to *R. helvetic*a and 1:128/1:128 to *R. felis* was used. The secondary antibody alone served as the negative control, together with serum from a healthy blood donor.

## Results

Of the 36 analysed patients, three (8.3%) were seropositive, with IgG titres equal to 1:128 (Table 1) against *R. helvetica*. An additional 14 patients (38.9%) had threshold IgG titres of 1:64, and 19 patients (52.8%) were seronegative (<1:64) for IgG antibodies. Concerning IgM antibodies, six patients (16.7%) had titres equal to or above 1:128, nine (25.0%) had titres equal to 1:64, and 21 (58.3%) were seronegative (<1:64). Two of the patients with IgG titres of 1:128 also showed a moderate increase (1:64) in IgM titres. Only one patient (No. 7) had a detectable IgG titre (1:64) against *R felis*.

**Table 1.** Results of serology (IFA and Western Blot) for *Rickettsia* and *Borrelia* in serum, selected treatment and sequelae among patients diagnosed with peripheral facial palsy

			Rickettsia – IFA		WB		Borrelia–EIA				
Pat No	Date of sampling (d-m)	Duration of symptoms (w/days)	IgG	IgM	IgG	IgM	IgG	IgM	viral assays (PCR)	Treat-ment	Sequelae
1	15-jan	12 w	1/128	1/64	pos	neg	180	<16	ND	P	0
2	09-jan	10 d	1/64	<1/64	neg	(pos)	<16	22	ND	P	0
3	12-jan	5 w	1/64	1/128	neg	pos	<16	<16	ND	P	0
4	14-jan	3 w	<1/64	<1/64	neg	neg	100	32	ND	P, AV	S
5	16-jan	6 d	1/64	<1/64	neg	neg	<16	<16	ND	P, dc	S
6	27-jan	5 w	1/64	<1/64	neg	(pos)	59	16	ND	NT	S
7	02-feb	36 w	<1/64	<1/64	neg	neg	<16	<16	ND	P	0
8	02-feb	10 d	1/64	1/256	neg	pos	140	39	ND	P, dc	S
9	04-feb	200 w	<1/64	1/64	neg	pos	<16	60	ND	NT	S
10	04-feb	12 d	<1/64	1/256	neg	pos	<16	<16	ND	NT	0
11	06-feb	13 d	<1/64	1/64	neg	pos	<16	17	ND	P	0
12	09-feb	19 w	1/64	1/64	neg	neg	33	<16	ND	NT	0
13	13-feb	6 d	<1/64	<1/64	neg	neg	<16	<16	ND	P	0
14	13-feb	6 d	<1/64	<1/64	neg	(pos)	<16	<16	ND	P	0
15	18-feb	6 w	1/64	<1/64	neg	neg	<16	<16	ND	P	0
16	24-feb	9 d	<1/64	<1/64	neg	neg	<16	<16	ND	P	0
17	24-feb	11 w	<1/64	1/128	neg	pos	<16	<16	ND	P, AV	0
18	24-feb	6 w	<1/64	<1/64	neg	neg	<16	<16	ND	P	S
19	27-feb	1 d	<1/64	<1/64	neg	neg	<16	<16	*	P, AV,	S
20	09-mars	9 w	1/64	1/128	neg	pos	<16	<16	ND	P	0
21	10-mars	5 w	<1/64	1/64	neg	neg	<16	<16	ND	P	0
22	10-mars	5 w	1/64	1/256	neg	pos	<16	47	ND	P, dc	0
23	11-mars	14 d	<1/64	<1/64	neg	(pos)	<16	<16	ND	P	S
24	26-mars	6 w	<1/64	<1/64	neg	neg	27	16	ND	P	0
25	07-apr	18 d	<1/64	<1/64	neg	neg	<16	<16	ND	P	S
26	09-apr	8 w	<1/64	<1/64	neg	neg	<16	<16	ND	P	0
27	22-apr	32 w	1/64	<1/64	neg	neg	<16	<16	ND	P, AV	0
28	29-apr	7 w	<1/64	1/64	neg	pos	<16	<16	ND	P	0
29	29-apr	11 d	1/64	<1/64	neg	neg	<16	18	ND	P	0
30	05-may	2 d	1/64	1/64	neg	(pos)	>200	78	ND	P	0
31	07-may	2 d	1/64	1/64	neg	(pos)	<16	<16	ND	P	0
32	11-may	8 w	1/128	<1/64	pos	neg	18	<16	ND	NT	0
33	15-may	4 w	1/128	1/64	pos	(pos)	19	<16	ND	NT	S
34	20-may	11 d	<1/64	<1/64	neg	neg	<16	<16	ND	P	0
35	12-juni	3 w	1/64	<1/64	(pos)	neg	<16	<16	ND	p	0
36	16-juni	4 w	<1/64	<1/64	neg	neg	34	<16	ND	P	S

AV = antiviral; S = sequelae

*varicella-zoster-virus DNA POS; HSV DNA neg

The corresponding findings for anti-*Borrelia* antibodies showed four patients (11.1%) with IgG antibodies against *Borrelia* at a titre level normally judged to be indicative of current infection (>100 RU/mL). Three of these patients also had an IgG and IgM response to *R. helvetica*. Another three had slight to moderate increases in IgG titre for *Borrelia* judged as past exposure. Two patients were seropositive only for IgM moderate titre levels and three had significant increases in both IgG and IgM. Two of these latter patients (No. 1 and 8) also had significant increases in IgG or IgM titres for *Rickettsia* spp. None of the patients had symptoms or clinical findings consistent with neuroborreliosis.

Patients with an IgG titre level of 1:128 were sampled 4 to 12 weeks after onset of FNP. In contrast, patients with an IgM response ≥ 1:128, were sampled 10 days to 11 weeks after onset.

Among the blood donors, three of 50 (6%) were seropositive for IFA, with a maximum level of antibody IgG titres of 1:64.

**Figure 1.**
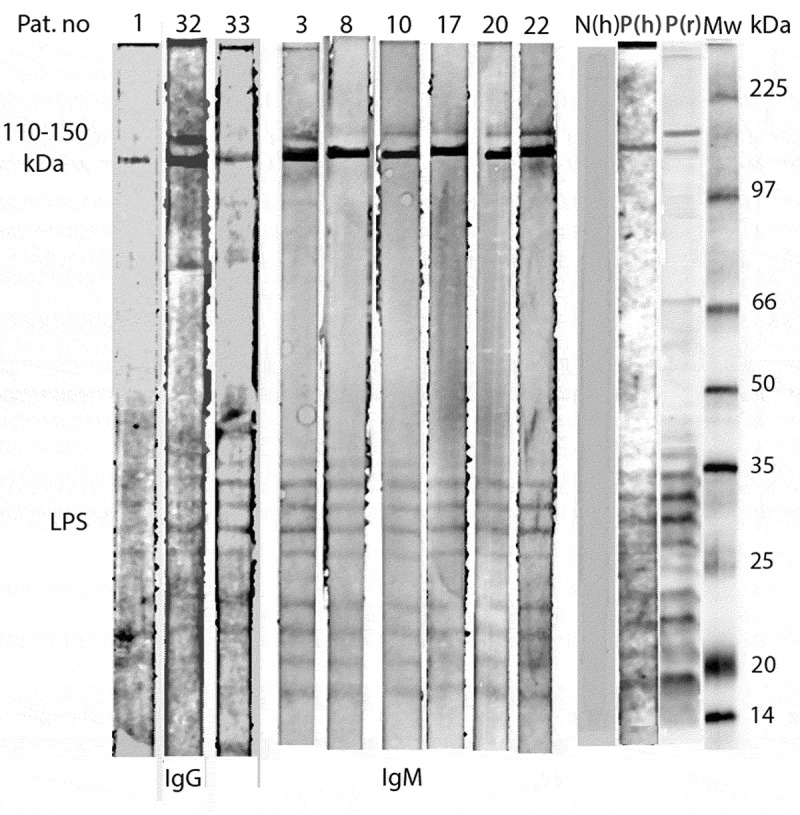
Western Blot analysis of IgG and IgM antibodies against *R. helvetica* whole cell antigen demonstrates the lipopolysaccaride (LPS) ladders and specific reactions against *R. helvetica* proteins in the 110–150-kDa region in serum for IgG for patients 1, 32 and 33 and for IgM for patients 3, 8, 10, 17, 20 and 22 in dilution 1:200. Lane P(h) demonstrates specific proteins and the LPS ladders reacting with a positive human serum and P(r) with a polyclonal rabbit antiserum. N(h) represent a negative human serum control

For 23 of 36 samples, the findings showed consistency between the IFA and the WB results for IgG antibodies, both negative and positive results, and for 29 of 36 samples for IgM antibodies. All three samples with an IgG titre level ≥ 1:128 in IFA showed uniformity with WB, but only one of 16 samples with a titre level of 1:64 was positive in Western blot. For IgM the equivalent correlation was the same for samples with titre level of 1:128, where six of six showed similar outcomes for both methods, as well as in seven of nine samples with an IgM titre level of 1:64 (Figure 1).

Thirty of 36 patients (83%) were treated with prednisolone during the first days of sickness; four of them received prednisolone in combination with antiviral medication or doxycycline treatment. Eleven patients (31%) showed persistent symptoms, ranging from paresis to mild muscular weakness in the affected area. Two of them had serological signs of *Borrelia* spp., while one had an elevated IgM titre for *Rickettsia* spp.

## Discussion

The present report shows that between 8.3% and 25% of patients with peripheral facial nerve palsy displayed serological findings indicating an association with a rickettsial infection. In patients with an IgG or an IgM titre in immunofluorescence equal to or higher than 1:128, assessed as active infection or early response, the specificity of the reaction was confirmed in Western Blot in 100% of the samples in which LPS bands were also clearly demonstrated. A 2-step approach was used, which gives a more reliable diagnostic result than a serological test alone does. Findings in patients with threshold IgG titres of 1:64, mostly without IgM response, probably represent past infection or exposure. Four of the patients (11.1%) had titres indicating current infection with *Borrelia* spp., and two of the four also showed serological evidence of a rickettsial infection, with IgG or IgM equal to or higher than 1:128, which might indicate co-infection [18,19]. Only one patient had detectable antibodies against *R. felis*, which is why the findings in the other patients may be assumed to represent infection with *R. helvetica*. The prevalence of *R. helvetica* in *Ixodes* ticks in Sweden is about 1.5–17%, and between 9% and 29% for *Borrelia* in different areas in Sweden [3,10,17]. Six percent of the 50 blood donors had low levels of rickettsia antibodies due to previous exposure. This can be compared with previous results showing that 2% of healthy blood donors in non-endemic areas were positive for *Borrelia* spp., compared to 9%-12% of individuals in endemic areas [19, 20,21,].

The current results are consistent with findings from a recent report in which 8.3% of patients had serological evidence of infection with *Rickettsia* spp. and symptoms of facial paralysis [9]. Note that patients who seroconverted in that survey showed an IgG titre (1:128) in sample two that corresponds to the present findings.

Patients with serologic evidence of infection or early response to infection, both for *Rickettsia* spp. and *Borrelia* spp., were equally distributed over the entire 6-month period. Two of the patients with Rickettsia IgM-antibodies had debuted with FNP 10 to 12 days before sampling while the other IgG or IgM positive patients debuted between 4–12 weeks earlier. Regarding LB, it is known that the time between the tick bite and nervous system symptoms may extend up to 12 weeks if the infection primarily occurs in the skin [22,23]. Similar phenomena have not been described for the onset of rickettsial infections, yet a tick-borne rickettsial infection is likely to be considered, as all patients in the present study had higher titres for *R. helvetica*, and only one patient had a threshold titre for *R. felis*.

About 11% of patients had serological findings that support a LB diagnosis, which corresponds to the frequency previously reported [7]. However, only one patient was suspected of having erythema migrans, and the others lacked objective signs in their medical history or clinical investigation for Lyme infection.

Corticosteroids during the first 5–10 days of FNP are the mainstay of medical treatment, and all but six patients were treated with them [24]. If there were signs of viral infection, antiviral drugs were also added; similarly, antibiotics were prescribed in cases of suspected borreliosis or serological evidence of Lyme disease. Eleven patients developed sequelae as a result of incomplete healing. They were found both among those who received treatment with prednisolone and among those who were treated with combination treatments. However, no clear correlation with seropositivity for *Borrelia* or *Rickettsia* could be seen.

The present study indicates that when FNP is displayed, it is equally common to find serological evidence of a rickettsial infection as it is to find evidence of Lyme borreliosis. Because the study was retrospective in nature – that is, was not conducted on paired sera and included a limited number of patients over a period of less than a year – it should be seen as a pilot study that needs to be followed up prospectively for a longer period and to include a larger number of patients. This would enable a more reliable assessment of the prevalence of rickettsia infection and FNP. These issues must be studied further, but though Sweden is an endemic area for *R. helvetica* and probably also for *R. felis*, it may be of importance to consider a rickettsial infection upon investigation and diagnosis of facial palsy.
